# An Unusual Presentation of Subacute Thyroiditis As Pyrexia of Unknown Origin With Normal Thyroid Functions

**DOI:** 10.7759/cureus.28807

**Published:** 2022-09-05

**Authors:** Muhammad Waqar, Chioma Okaneme, Sripriya Rasthapuram, Muhammad Aadil, Tamar Saeed

**Affiliations:** 1 Internal Medicine, Dudley Group NHS Foundation Trust, Dudley, GBR; 2 Internal Medicine, Russells Hall Hospital, Dudley, GBR; 3 Diabetes and Endocrinology, Russells Hall Hospital, Dudley, GBR

**Keywords:** thyroid function, ct tap, pet scan, subacute thyroiditis, pyrexia of unknown origin

## Abstract

Subacute thyroiditis is a rare condition believed as immune-mediated inflammation of the thyroid gland that frequently manifests after a viral upper respiratory tract infection. A 52-year-old South-Asian female patient presented to Same Day Emergency Care (SDEC) with feeling unwell and sore throat. Moreover, she reported low-grade fever, fatigue, headache, and breathlessness on exertion for the past five weeks. She had a past medical history of gastroesophageal reflux disease. She had no associated cough, rigors, chills, urinary symptoms, night sweats, or weight loss. There was no history of recent travel abroad. On examination, she was tachycardic; however, there was no lymphadenopathy, palpable thyroid gland, skin rash, or signs of infective endocarditis. Routine blood analysis showed elevated erythrocyte sedimentation rate (ESR), C-reactive protein (CRP), and normal white blood cell count. Her thyroid stimulating hormone (TSH) was normal. Chest X-ray and echocardiogram were unremarkable. She was prescribed clarithromycin with no effect. After inconclusive results of the routine investigation and no response to antibiotics, a computed tomography (CT) scan of the thorax, abdomen, and pelvis (TAP) was performed, which revealed a thickened thyroid isthmus. Positron Emission Tomography (PET) scan revealed bulky appearances of the thyroid gland with diffuse increased uptake suggestive of thyroiditis. Prednisolone 30mg daily was prescribed, which was later reduced by 5mg weekly for six weeks. The patient showed improvement in symptoms, and normal ESR and CRP were achieved.

## Introduction

Subacute thyroiditis (SAT) is a rare condition believed as immune-mediated inflammation of the thyroid gland that frequently manifests after viral upper respiratory tract infections like mumps, influenza, and other respiratory viruses. It is usually diagnosed clinically and then confirmed biochemically, i.e., thyroid functions tests (TFTs) showing elevated triiodothyronine (T3) and thyroxine (T4) and suppressed thyroid stimulating hormone (TSH) in addition to raised inflammatory markers like ESR and CRP [1]. The usual presentation of SAT is swollen and painful neck due to an inflamed thyroid gland. Other symptoms are voice hoarseness, difficulty and pain in swallowing, fatigue, and fever [2]. The SAT is usually a self-limiting condition, and routinely symptomatic treatment is needed. Nonsteroidal anti-inflammatory drugs are prescribed for pain control and corticosteroids to resolve inflammation. Prednisolone has better efficacy in the treatment of SAT [3].

To the best of our knowledge, based on a thorough review of the literature using PubMed, Medline, and Google Scholar, no such case with an unusual presentation has been published in the literature. This case report will provide clinicians with information regarding atypical presentations of SAT and can lead to a proper diagnosis of the patient.

## Case presentation

A 52-year-old South-Asian female patient presented to Same Day Emergency Care (SDEC) with a feeling of generally unwell and sore throat. She had a past medical history of gastro-oesophageal reflux disease. She reported a five-week low-grade fever (>38.3°C), which occurs mainly in the evenings with fatigue, and sometimes experiences breathlessness on exertion. She had consulted her general practitioner (GP) and was prescribed clarithromycin for suspected respiratory tract infection, with no effect. There was no history of cough, rigors, chills, urinary symptoms, night sweats, weight loss, or recent travel abroad. The examination was unremarkable, except for tachycardia, with a heart rate of 106 beats per minute. There was no lymphadenopathy, palpable thyroid gland, skin rash, or stigmata of infective endocarditis. Routine blood analysis showed elevated erythrocyte sedimentation rate (ESR: 110 mm/hr), elevated C-reactive protein (CRP: 78 mg/L), normal white blood cells, decreased hemoglobin (109g/L), and normal TSH (0.56 miu/L). Chest X-ray and electrocardiogram were unremarkable.

 Given many weeks’ history of intermittent pyrexia, ethnicity, and raised ESR, the provisional diagnosis of tuberculosis or likely vasculitis was made. Further investigations, including blood and urine cultures, vasculitis screen (Antineutrophil Cytoplasmic Antibody, Antinuclear Antibody, Immunoglobulins), viral serology (including HIV), procalcitonin, and T-spot were done.

**Figure 1:**
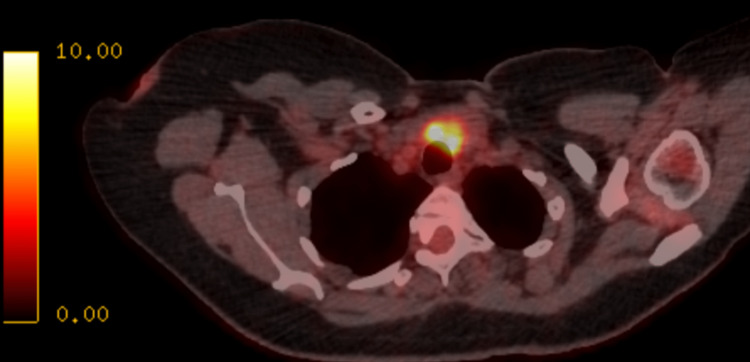
Shows bulky thyroid gland tissue on PET scan suggestive of thyroiditis 876x422mm (38 x 38 DPI)

The patient was started on a trial of ciprofloxacin for a suspected urinary tract infection. These investigations were inconclusive. On the first review appointment, her symptoms worsened with a new frontal headache, vomiting, odynophagia, and reduced appetite but no neck stiffness. Possible meningitis was suspected, and a CT scan of the head and lumbar puncture were performed, which was inconclusive. Therefore, antibiotics were discontinued, and a positron emission tomography (PET) scan was requested. On the second review appointment, while the patient was awaiting a PET scan, a CT scan of the thorax, abdomen, and pelvis (TAP) was performed, which showed thickened thyroid isthmus. However, the rest of the study was unremarkable. The PET scan later revealed bulky appearances of the thyroid gland with diffuse increased uptake, suggestive of thyroiditis (figure 1).

However, the patient had no clinical features of hyperthyroidism, and the thyroid functions were normal, i.e., TSH was 0.46 miu/L, free T4 was 15.1 pmol/L, and T3 was 3.8 pmol/L. Immunology tests, including thyroid peroxidase (TPO) and Thyrotropin receptor antibodies, were normal (15 IU/ml and 1.77 IU/L, respectively). Based on the PET scan, the diagnosis of subacute thyroiditis as the cause of pyrexia of unknown origin (PUO) was reached. She was commenced on prednisolone 30mg daily, which was later reduced by 5mg per week for six weeks. On day 5 of steroids, the patient reported improvement in symptoms, and repeat blood tests showed improved ESR (46 mm in 1st hour) and CRP (2 mg/L).

## Discussion

Thyroiditis is a thyroid gland inflammation classified based on etiology, pathology, or clinical presentation. It may be painful, which includes subacute thyroiditis and suppurative thyroiditis, or painless, in which there is thyroid dysfunction or enlargement without any acute inflammation [2]. Although subacute thyroiditis is the commonest form of painful thyroiditis [4], it is rare (5% of thyroid disorders). It is usually seen among the middle age group, predominantly in females (80%) [5]. Thyroid gland pain is the commonest symptom (90%) found in subacute thyroiditis but may be absent in up to 10% of cases, as in our case. Similarly, in most cases, hyperthyroidism is present in contrast in our patient, who remained euthyroid [6].

Moreover, long history of fever with raised inflammatory markers and no response to two courses of antibiotics directed a thorough work-up for pyrexia of unknown origin [7-8]. As part of initial screening, CT TAP reported thyroid swelling, which PET Scan later confirmed. Nonsteroidal anti-inflammatory drugs and steroids are commonly used as initial therapy in symptomatic patients [9]. The patient returned to her normal clinical and biochemical baseline after six weeks of treatment.

Kumar et al. reported a case of subacute thyroiditis, a 55 years insulin-dependent diabetic patient who presented with a history of fever of one-month duration. Their case had elevated ESR, CRP, and abnormal thyroid function tests [10]. On the other hand, our case had normal TFTs.

Dalugama [4] published a case report of a 42-year-old Sri Lankan male who presented with a three weeks history of fever and had mild tenderness over his neck with cervical lymphadenopathy with no thyrotoxic symptoms or signs. The laboratory investigation showed low thyroid-stimulating hormone and high T3 and T4. Fine needle aspiration biopsy confirmed Subacute thyroiditis. This report is also different because of normal TFTs in our case.

We treated our case with NSAIDs and steroids. No universal guidelines exist so far for the treatment of SAT, and a low-dose steroid of 10mg prednisolone is usually considered an effective treatment for SAT [11,12].

## Conclusions

This case demonstrates that subacute thyroiditis though one of the causes of PUO, rarely presents with normal thyroid function tests, which should also be considered in such patients. We do not advocate PET scans to look for subacute thyroiditis as the cause of PUO. However, routinely available radiological investigations like a thyroid ultrasound scan would help diagnose such cases. Therefore, SAT should be considered in differential diagnoses of patients presenting with PUO with no classical features of thyrotoxicosis.
